# Clonal amplification of *Fasciola hepatica* in *Galba truncatula*: within and between isolate variation of triclabendazole-susceptible and -resistant clones

**DOI:** 10.1186/s13071-018-2952-z

**Published:** 2018-06-26

**Authors:** Jane E. Hodgkinson, Krystyna Cwiklinski, Nicola Beesley, Catherine Hartley, Katherine Allen, Diana J. L. Williams

**Affiliations:** 1 Veterinary Parasitology, Dept Infection Biology, Institute of Infection and Global Health, University of Liverpool, Liverpool, L69 7ZJ UK; 2 School of Biological Sciences, Medical Biology Centre, Queen’s University Belfast, Belfast, BT9 7BL UK

**Keywords:** *Fasciola hepatica*, *Galba truncatula*, Triclabendazole resistance, Clonal isolate, Isolate variation

## Abstract

**Background:**

*Fasciola hepatica* is of worldwide significance, impacting on the health, welfare and productivity of livestock and regarded by WHO as a re-emerging zoonosis. Triclabendazole (TCBZ), the drug of choice for controlling acute fasciolosis in livestock, is also the drug used to treat human infections. However TCBZ-resistance is now considered a major threat to the effective control of *F. hepatica*. It has yet to be demonstrated whether *F. hepatica* undergoes a genetic clonal expansion in the snail intermediate host, *Galba truncatula*, and to what extent amplification of genotypes within the snail facilitates accumulation of drug resistant parasites. Little is known about genotypic and phenotypic variation within and between *F. hepatica* isolates.

**Results:**

Six clonal isolates of *F. hepatica* (3× triclabendazole-resistant, TCBZ-R and 3× triclabendazole-susceptible, TCBZ-S) were generated. Snails infected with one miracidium started to shed cercariae 42–56 days post-infection and shed repeatedly up to a maximum of 11 times. A maximum of 884 cercariae were shed by one clonally-infected snail (*Fh*LivS1) at a single time point, with > 3000 clonal metacercariae shed over its lifetime. Following experimental infection all 12 sheep were FEC positive at the time of TCBZ treatment. Sheep infected with one of three putative TCBZ-S clones and treated with TCBZ had no parasites in the liver at *post-mortem*, whilst sheep each infected with putative TCBZ-R isolates had 35–165 adult fluke at *post-mortem*, despite TCBZ treatment. All six untreated control animals had between 15–127 parasites. A single multi-locus genotype was reported for every fluke from each of the six clonal isolates. Adult *F. hepatica* showed considerable variation in weight, ranging from 20–280 mg, with variation in weight evident within and amongst clonal isolates.

**Conclusions:**

A genetic clonal expansion occurs within *G. truncatula*, highlighting the potential for amplification of drug resistant genotypes of *F. hepatica*. Variation in the weight of parasites within and between clonal isolates and when comparing isolates that are either susceptible or resistant to TCBZ represent inherent variation in liver fluke and cannot be attributed to their resistance or susceptibility traits.

## Background

*Fasciola hepatica* is a trematode parasite of worldwide significance. A commonly diagnosed helminth parasite in sheep and cattle it is increasing in prevalence and spreading into new areas [1, 2] and is regarded by the WHO as a re-emerging zoonosis, with an estimated 17 million people at risk of infection [3]. It has a two-host life-cycle involving a mammalian definitive host, typically sheep and cattle, and a molluscan intermediate host, predominantly *Galba truncatula* in the UK and across western Europe*.* Following embryonation of *F. hepatica* eggs shed in the faeces of an infected host, the miracidia hatch and infect the snail, where parasite amplification occurs prior to shedding of multiple cercariae ~6–8 weeks post-infection. These cercariae encyst on vegetation as metacercariae; the infective stage that is subsequently ingested by the definitive host. On ingestion of metacercariae the newly excysted juvenile (NEJ) stage migrates through the liver and can cause disease known as acute fasciolosis, a significant cause of morbidity and mortality in livestock, particularly sheep. Temperature and rainfall are the principle determinants affecting the life-cycle and hence the prevalence and intensity of *F. hepatica* infection [2]. It is suggested that climate change is at least partly responsible for the increase in the prevalence of *F. hepatica* [4] and is thought to exacerbate the financial and welfare impact of fasciolosis on livestock production [5].

Triclabendazole (TCBZ), a key compound in the control of acute fasciolosis in livestock due to its efficacy against NEJ as early as two days post-infection [6], is also the drug of choice to treat human fasciolosis. The heavy reliance on TCBZ to treat sheep and cattle at frequent intervals has resulted in the emergence of TCBZ-resistance first in Australia and subsequently in many other countries [7–9]. It is now considered a major threat to the effective control of *F. hepatica* worldwide [10, 11]. The genetic and molecular basis of TCBZ resistance remains unknown. Linkage mapping for drug resistance has proved highly successful for protozoan parasites, for example malaria and *Eimeria* [12], and for trematodes such as *Schistosoma mansoni* [13, 14]. These mapping exercises rely on access to a number of specific resources; a high quality genome assembly and linkage map [15] and the availability of clonal isolates. Significant advances have been made recently with publication of the first draft of the *F. hepatica* genome in 2015 [16] and work is underway to enhance its assembly. To date the majority of TCBZ resistance studies have been conducted on laboratory isolates, for example, the TCBZ-susceptible (TCBZ-S) Fairhurst and Cullompton isolates and TCBZ-resistant (TCBZ-R), Sligo and Oberon isolates [17, 18]; which were isolated from the field more than a decade ago [19, 20]. The need to routinely confirm the susceptibility of isolates used in drug resistance and drug development trials was highlighted recently [21]. At the genetic level, all currently available fluke isolates are either not characterised or at best are only partially genetically defined. A source of genotypically characterised clonal lines, phenotypically defined for their sensitivity to key drugs like TCBZ, is essential to the success of forward genetic approaches that have proved so successful in mapping drug resistance loci in other parasites [14, 21, 22].

Here, we report the production of six clonal isolates by experimental infection of *G. truncatula* with a single miracidium derived from one of five field isolates and one laboratory maintained isolate of *F. hepatica* (Shrewsbury isolate, Ridgeway Research Ltd). Clonal isolates were phenotypically defined for their sensitivity to TCBZ by experimental infection in sheep and subsequent treatment with TCBZ at the recommended dose rate of 10 mg/kg. Genotyping of each isolate was carried out using a *F. hepatica* microsatellite panel [23]. These neutral markers were used to confirm the capacity for genetically clonal amplification of *F. hepatica* within *G. truncatula* and provided a multilocus genotype (MLG) with which to uniquely identify each clonal isolate.

## Methods

### Source of eggs for *Fasciola hepatica* clonal isolates

Six clonal isolates of *F. hepatica*, 3× TCBZ-R and 3× TCBZ-S, were generated. For the TCBZ-R isolates, three field isolates of *F. hepatica* were sourced from naturally infected sheep with a history of TCBZ treatment: *Fh*LivR1, Northwest England, UK; *Fh*LivR2, South Wales, UK and *Fh*LivR3, Northwest England, UK (a location distinct from *Fh*LivR1). For the TCBZ-S isolates, two field isolates were sourced from naturally infected sheep not previously exposed to TCBZ; *Fh*LivS2, Northwest England, UK and *Fh*LivS3, Southwest England, UK. An additional isolate, *Fh*LivS1, was sourced from cattle experimentally infected with the laboratory maintained TCBZ-S Shrewsbury isolate (Ridgeway Research Ltd, UK). Eggs were recovered from the gall bladder of infected sheep/purged from *F. hepatica* adult parasites at *post-mortem* (*Fh*LivS1, *Fh*LivR1 and *Fh*LivR3) or isolated from faecal samples using a standard sedimentation method (*Fh*LivS2, *Fh*LivS3, *Fh*LivR2).

### Maintenance and experimental infection of *G. truncatula* and production of *F. hepatica* metacercariae

*Galba truncatula* snail stocks were maintained on pans of clay mud and fed on a diet of *Oscillatoria* spp. algae. Both the snails and algae were maintained at a controlled temperature of 22 °C. For experimental infection of snails, *F. hepatica* eggs were embryonated at 27 °C in the dark for 14 days followed by exposure to a direct light source to stimulate hatching of miracidia. A single miracidium was co-incubated with an individual snail of approximately 4 mm in height in each well of a 48-well plate in ~200 μl of H_2_O + ~100 μl algae and incubated for a minimum of 4 h. Following infection, snails were maintained on mud pans and fed every 2–3 days. At 6 weeks post-infection (wpi), individual snails were sealed in visking tubing containing H_2_O and stimulated to shed cercariae by exposing snails to a drop in temperature to 12 °C for 30 min followed by a slow return to RT under a light source over a period of several hours. They were then left overnight to allow metacercariae to encyst on the visking tubing. Metacercariae were subsequently stored at 4 °C. Individual snails were shed repeatedly and metacercariae from each individual snail were pooled prior to infection of sheep.

### Experimental infection of sheep and recovery of adult parasites at *post-mortem*

Typically, experimental infections were carried out using lambs ~3 months of age that had no access to pasture. Animals were housed in group conditions in an indoor facility and fed on growing sheep concentrate pellet with hay and water provided *ad libitum*. Lambs were inspected at least daily for the duration of the sampling period. Prior to infection lambs were confirmed free of *F. hepatica* infection by antibody detection ELISA using *F. hepatica* ES antigens ELISA [24] and faecal egg count (FEC) using a standard sedimentation technique. Infections were performed by oral administration of metacercariae to two sheep per isolate using a dose of ~200 clonal metacercariae per sheep. The viability of metacercariae was assessed prior to infection by visualising flame cells, if no flame cells were seen the metacercariae were considered to be non-viable. One sheep from each pair was treated with TCBZ at the recommended dose rate of 10 mg/kg, whilst the other sheep remained as an untreated control. All sheep were euthanized 10 days post-TCBZ treatment (time of euthanasia was different for each clone and ranged from 17–24 wpi). Infection status was monitored weekly by ELISA from four weeks prior to infection until *post-mortem* or by FEC from, pre-infection and then from eight wpi until necropsy. At *post-mortem* liver flukes were manually recovered directly by dissection from the bile ducts or following slicing of the liver and incubation in PBS for 2 h at 37 °C. Eggs were harvested from adult parasites purged by incubation in 1–2 ml of Dulbecco’s Modified Eagle’s Media (DMEM; Sigma-Aldrich, Dorset, UK) for a minimum of 2 h at 37 °C. The parasites were then removed from the culture medium, whole parasites were weighed and all fluke material (including partial fluke) was snap frozen in liquid nitrogen. Purged eggs were stored at 4 °C; batches were periodically embryonated as described above and passaged through snails and sheep to maintain clonal lines.

### Genotyping of clonal isolates

The entire adult fluke or a section of adult fluke near the anterior sucker, representing 20 mg of tissue was used for DNA extraction. Genomic DNA extraction was carried out using the DNeasy Blood and Tissue Kit (for 20 mg tissue) or Genomic tip 100G (for whole adult fluke) (both Qiagen, Manchester, UK), according to the manufacturer’s instructions. For the majority of samples this was followed by an ethanol precipitation and re-suspension of DNA in a volume of 60 μl TE (pH 8.0). All DNA concentrations were calculated using the Quant-iT^TM^ PicoGreen® dsDNA assay kit (Life Technologies, Thermo Fisher Scientific, Rugby, UK). Microsatellite PCR and sequencing was essentially carried out as described [23, 25] by employing the multiplex protocol using the Type-it Microsatellite PCR kit (Qiagen, UK) according to the manufacturer’s instructions and 1 μl (10 ng) of adult genomic DNA template. PCR products were analysed by agarose gel electrophoresis (2% gel) using SYBR® Safe DNA stain (Life Technologies). If a positive result was obtained by gel electrophoresis, the PCR products were diluted 25 or 50 fold and 1 μl of this dilution multiplexed in Hi-Di Formamide (8.8 μl; Life Technologies) with GeneScan LIZ500 size standards (0.2 μl; Life Technologies), prior to sequencing using a 3100 Genetic Analyzer capillary electrophoresis system (Life Technologies) and Peak Scanner v1.0 software.

## Results

### Clonal amplification of *Fasciola hepatica* in *Galba truncatula*

Five clonal isolates were derived using populations of eggs from naturally infected UK sheep flocks, from one of several geographical locations (Table 1). Low rates of infection in snails and high mortality of infected snails commonly occurs in experimental systems, hence, to generate the six clonal isolates multiple individual snail:miracidium infections were set up for each population; with a total of between 30–40 snails clonally infected per isolate. Infected snails were first detected to be shedding cercariae from 42–56 days post-infection (dpi). Although some snails shed once and died, the majority (75–89.6%) continued to shed cercariae multiple times up to a maximum of 11 times. A snail infected with one miracidium can generate large numbers of cercariae, with one snail producing > 3000 clonal metacercariae (Table 2). Infected snails shed vastly different numbers of cercariae over their lifetime with a maximum of 884 cercariae shed by any one snail at a given time-point (data not shown). The snail shedding the largest number of cercariae was selected from each population and a subset of ~400 metacercariae derived from this clonally-infected snail was used to infect the two sheep for each clonal isolate (~200 metacercariae per sheep). A single MLG was reported for every fluke from each of the six isolates (Table 4), uniquely identifying each clone. The presence of fluke of just a single genotype in each animal (or pair of animals infected with metacercariae derived from the same snail) confirmed a genetic clonal expansion had occurred in the snail and a clonal infection was established in the definitive host.

**Table 1:** Details of field samples used to generate six *Fasciola hepatica* clonal isolates

Clonal isolate	Origin of *F. hepatica* eggs	Geographical location of farm	History of TCBZ exposure
*Fh*LivR1	Purged from adult parasites recovered at PM (AHVLA)	North West England	Frequent use of TCBZ, treated with TCBZ 28 days prior to PM and 2 days prior to PM. Sheep died 2 days post-treatment
*Fh*LivR2	Isolated from faecal sample taken 21 days post-TCBZ treatment	South Wales	Population of *F. hepatica* showing 98.2% reduction based on FECRT^a^
*Fh*LivR3	Purged from adult parasites recovered at PM (AHVLA)	North West England (not same farm as R1)	History of TCBZ treatment failure on farm, last exposed to TCBZ 4 months prior to PM
*Fh*LivS1	Shrewsbury laboratory isolate, commercially available TCBZ susceptible population (Ridgeway Research Ltd, UK)	Isolated 2006 from West England	Population of *F. hepatica* showing TCBZ efficacy^b^ of 97% in sheep (*n* = 9), Ridgeway Research Ltd (pers. comm.)
*Fh*LivS2	Isolated from faecal samples from organic sheep farm	North West England (not same farm as R1 or R3)	None
*Fh*LivS3	Isolated from faecal samples from organic sheep farm	South West England	Population of *F. hepatica* showing TCBZ efficacy^b^ of 97.2% in sheep (*n* = 12, data not shown)

^a^TCBZ-S based on criteria for faecal egg count reduction test (FECRT) [45]

^b^TCBZ % efficacy defined based on critical test performed at 10 days after treatment and using the following calculation: % efficacy = (mean of *F. hepatica* in control group – mean of *F. hepatica* in treated group/ mean of *F. hepatica* in control group) × 100 [46]

*Abbreviations*: *PM post*-*mortem*, *AHVLA/APHA* Animal Health Veterinary Laboratory Agency/Animal and Plant Health Agency, *TCBZ* triclabendazole

**Table 2:** Experimental infection of sheep for production of *Fasciola hepatica* clonal isolates

Clonal isolate	Total no. of cercariae shed by a single snail used to derive clonal infections	No. of metacercariae used for infection	Timepoint of PM (wpi)	No. of adult flukes recovered at PM^a^
*Fh*LivR1	931	150	18	70
		220	18	165 (T)
*Fh*LivR2	553	193	18	31
		200	18	48 (T)
*Fh*LivR3	812	256	18	35 (T)
*Fh*LivS1	3200	209	19	15
		224	19	0 (T)
*Fh*LivS2	> 417^b^	215	24	113
		202	24	0 (T)
*Fh*LivS3	1166	200	17	127
		200	17	0 (T)

^a^Two sheep were infected for each isolate and the dose of metacercariae given to each sheep is shown. Patency of infection was confirmed by faecal egg count and one sheep from each pair was treated with 10 mg/kg triclabendazole (T) 10 days prior to *post-mortem* (PM) with the exception of *Fh*LivR3 where one sheep had to be euthanized during the course of the experiment. The number of adult flukes recovered from each sheep at PM is shown

^b^Actual number not calculated

*Abbreviations*: *wpi* weeks post infection, *PM post-mortem*

### Phenotyping of TCBZ-susceptible and TCBZ-resistant clonal isolates of *Fasciola hepatica*

All three populations from which TCBZ-R clonal isolates were generated, were from cases referred by the farmer to the Veterinary Investigation Centre, Camarthen, Wales, UK (AHVLA, now called the Animal and Plant Health Agency, APHA); they were cases of suspected TCBZ resistance, due to therapeutic drug failure or death of sheep post-TCBZ treatment.

Prior to experimental infection all animals recorded negative FEC values and for those animals tested by ELISA (*Fh*LivS1-S3) low percent positivity (PP) values were recorded (0.2–6.5). A single dose of between 150–256 metacercariae from a single snail exposed to one miracidia was administered to two animals (see Table 2). All animals were seropositive by 4 wpi (ELISA PP values ranged from 18.3–54.4) and were FEC positive by 10 wpi and eggs were consistently shed by all sheep until the day of treatment. The sheep recording the highest egg count was treated with TCBZ at 10 mg/kg whilst the other remained untreated (Table 3). All 12 sheep were FEC positive on the day of treatment (Table 3) consistent with the presence of egg producing adult parasites at the time of TCBZ treatment. Sheep infected with *Fh*LivS1, S2 or S3 and treated with TCBZ had no adult liver fluke present at *post-mortem*, whilst TCBZ treated sheep infected with *Fh*LivR1, R2 or R3 had fluke burdens ranging from 35–165 (Tables 3, 4). All six untreated control animals had liver fluke burdens with between 15–127 parasites per sheep (Table 2). This confirmed the resistant or susceptible phenotype of each isolate. The adult *F. hepatica* from each isolate showed considerable variation in the size of fluke both within and between clonal isolates, although it was not practical to measure the size of individual adult fluke. This was reflected in their weight; the larger the fluke the greater the weight. A minimum weight of 20 mg (*Fh*LivR1 isolate) and a maximum weight of 280 mg (*Fh*LivS3) was found. This variation was evident not only between clonal isolates but within each clonal isolate (Table 4).

**Table 3:** Faecal egg count and adult parasite burden at *post*-*mortem* for six *Fasciola hepatica* clonal isolates. Two sheep were infected for each clonal isolate, patency of infection was confirmed by faecal egg count (FEC), expressed as eggs per gram (epg). One sheep from each pair was treated with 10mg/kg triclabendazole, the other sheep was left untreated. After 10 days all sheep were euthanised and adult liver fluke were enumerated *post-mortem*

Clonal isolate	FEC (epg) on day of treatment		Number of adult liver flukes present *post*-*mortem*	
	TCBZ+	TCBZ-	TCBZ+	TCBZ-
*Fh*LivR1	381	137	165	70
*Fh*LivR2	324	50	48	31
*Fh*LivR3^a^	186	–	35	–
*Fh*LivS1	6	2	0	15
*Fh*LivS2	146	57	0	127
*Fh*LivS3	89	73	0	113

^a^*Fh*LivR3, one sheep (TCBZ-) had to be euthanized for non-fluke related illness during the course of the experiment

*Abbreviations*: *epg* eggs per gram, *FEC* faecal egg count; TCBZ+, treatment with 10 mg/kg triclabendazole; TCBZ-, no treatment with 10 mg/kg triclabendazole

**Table 4:** Phenotypic and genotypic characteristics of *Fasciola hepatica* clonal isolates

	No. of whole adult flukes recovered at PM	Mean weight of adult flukes ± SD (mg)	Median weight of adult flukes (mg)	Range of weights recorded for adult flukes (mg)	No. of adult flukes genotyped	No. of MLG observed
*Fh*LivR1	70	108 ± 22	110	30–150	70	1^a^
	165 (T)	83 ± 28	80	20–160	165	1^a^
*Fh*LivR2	31	86 ± 16.00	90	50–130	31	1^b^
	48 (T)	98 ± 20	100	60–140	48	1^b^
*Fh*LivR3	35 (T)	102 ± 30	110	40–160	35	1
*Fh*LivS1	15	177 ± 27	180	90–210	15	1
	0 (T)	–	–	–	–	–
*Fh*LivS2	105^c^	148 ± 25	150	80–230	110	1
	0 (T)	–	–	–	–	–
*Fh*LivS3	112^d^	129 ± 22	130	80–280	126	1
	0 (T)	–	–	–	–	–

^a^Same genotype in both animals

^b^Same genotype in both animals

^c^Plus an additional 8 partial parasites

^d^Plus an additional 15 partial parasites

*Abbreviations*: *FhLiv* clonal isolate of *Fasciola*, *R* resistant to triclabendazole, *S* susceptible to triclabendazole, (*T*) treated with 10mg/kg triclabendazole 10 days prior to PM, *PM post-mortem*, *MLG* multilocus genotype based on a panel of 15 microsatellite markers

### Genotyping of TCBZ-susceptible and TCBZ-resistant clonal isolates of *Fasciola hepatica*

A high level of genetic differentiation was observed between isolates. The MLG for each isolate was distinct and a number of alleles were unique to each isolate, in particular loci Fh_2, Fh_4, Fh_5, Fh_6 and Fh_11 were represented by a different genotype for each isolate (Table 5).

**Table 5:** Unique multilocus genotypes for each of the six clonal isolates

	Locus^a^														
Clonal isolate	Fh_1	Fh_2^b^	Fh_3	Fh_4^b^	Fh_5^b^	Fh_6^b^	Fh_7	Fh_8	Fh_9	Fh_10	Fh_11^b^	Fh_12	Fh_13	Fh_14	Fh_15
*Fh*LivR1	08/10	08/47	07/08	18/20	31/31	33/36	13/13	11/16	07/07	09/11	08/08	07/10	08/11	11/19	14/14
*Fh*LivR2	08/08	08/45	08/08	16/18	24/24	14/33	11/11	11/16	06/07	09/15	07/14	10/15	08/11	17/18	09/14
*Fh*LivR3	11/11	14/19	07/08	16/20	27/32	30/32	11/12	12/12	07/07	09/11	11/12	15/16	08/08	18/18	09/14
*Fh*LivS1	08/08	18/19	07/09	18/23	24/32	31/33	12/12	14/14	06/07	10/16	12/15	10/10	08/08	08/08	09/14
*Fh*LivS2	06/06	08/08	07/08	14/20	27/31	14/16	12/12	11/19	06/07	06/11	08/15	10/10	08/16	17/19	14/14
*Fh*LivS3	10/16	14/15	07/08	16/19	27/30	16/29	13/13	12/12	07/07	11/14	13/13	05/10	08/11	08/08	09/14

^a^Alleles are identified by the number of microsatellite repeats. The two alleles of each genotype are separated by a /

^b^Loci where unique genotypes were recorded for each isolate

## Discussion

### Clonal isolates allow us to better understand *Fasciola hepatica* biology

To date, no study has analysed *F. hepatica* parasites at the molecular level following their expansion within the snail intermediate host. The presence of multiple adult parasites of the same MLG, within a host exposed to metacercariae from a single snail infected with just one miracidium, is consistent with the clonal amplification of *F. hepatica* within *G. truncatula*. Studies on closely related trematodes, including *Clonorchis sinensis*, *Opisthorchis viverrini* and *Schistosoma* spp., have used molecular tools to demonstrate a genetic clonal amplification occurs within the snail intermediate host and have shown that mitotic recombination events can occur [26–28]; however, based on our analysis here there is no evidence to support mitotic recombination in *F. hepatica*.

It is difficult to compare published studies of snail infections with *F. hepatica*, given that husbandry and diet can affect cercarial output [29, 30], but the experimental infection of snails performed here adds valuable insights into the capacity of snails to shed cercariae. The time from when snails were exposed to miracidia to the first observation of cercarial shedding is similar to previous studies [31–33]. However, the period of cercarial shedding by experimentally infected snails exceeds that reported elsewhere [31–34], but is similar to that of Hodasi et al. [35], where snails were fed the same diet (*Oscillatoria* spp.). The mean number of metacercariae produced by snails infected with a single miracidium has been reported as 114.9 (SD: 80.3) [32], which is lower than observed with our snails, probably due to the extended period over which our infected snails shed cercariae. As with other studies we observed that some snails are ‘super-shedders’ producing thousands of metacercariae [35].

Gene flow is driven by multiplication and recombination events in the intermediate and definitive hosts, respectively. The genetic clonal expansion of *F. hepatica* in snails has the potential to reduce diversity of *F. hepatica* in the definitive host, particularly when the typical prevalence of infection in snails in the field is low (~5%) [36–38]. However, it has been shown that, in naturally infected sheep and cattle, *F. hepatica* exists as a panmictic population with high levels of diversity in populations infecting the definitive host [25]. It is not known to what extent *F. hepatica* undergoes self and cross-fertilisation and how this might influence gene flow, or whether *F. hepatica* undergoes multiple mating events. Previously, we have shown that the selfing rate of adult liver fluke infection in the definitive host is no higher than 2% [25], which supports the current hypothesis that cross-fertilization predominates. In future, these clonal isolates, each identifiable with a unique MLG, constitute a valuable resource with which to explore gene flow and reproductive biology of the liver fluke life-cycle stages in vivo. Each of the six isolates can be tracked through infection in the snail and definitive host. Progeny (eggs and metacercariae) derived from mating between two parasite isolates, each with a distinct MLG, can be genotyped to identify if cross- or self-fertilization predominates in the definitive host and explore parasite mating behaviour.

### Variation within and between clonal isolates of *Fasciola hepatica*

The fact that each clonal isolate, irrespective of drug resistance status, has a unique and distinct genetic profile is not surprising given previous work on whole-genome sequence variation [16]. What is of particular interest here is phenotypic variation within a clonal isolate. In naturally infected hosts mature, adult fluke differ considerably in weight, ranging from 40–280 mg, mean 130 ± 4.36 mg (of 205 liver fluke sampled from 26 sheep livers, data not shown). The impact of the host environment on parasite development, size and intestinal position has been demonstrated for the parasitic nematode *Strongyloides ratti* [39]. This study has shown that clonal liver fluke, with identical MLG, can differ greatly in their weight (Table 4). This reveals how much the host environment influences the growth of parasites in the liver; for example, there is the potential for significant variation in the time taken to excyst, to exit the intestine and to enter liver tissue and migrate to the bile ducts. This variation is important, because size, along with specific morphological characteristics, is often used as a means of species-specific identification for *Fasciola* spp. [40]. Any/all of these variables could also influence the sizes of worms recorded. Regardless of what factors influence phenotypic variation, such variation is an important consideration when comparing isolates. For example, here it is not possible to attribute differences between isolates to resistance or susceptibility to TCBZ, because variation is apparent even when comparing two different isolates susceptible (or resistant) to TCBZ. This highlights that drug resistance studies on highly heterogeneous populations need to distinguish between the impact of standing genetic variation and that of genetic mutations responsible for conferring resistance traits. It is worth noting that a mutation in a PGP gene in a small number of TCBZ-resistant liver fluke in Northern Ireland [41] was not detected in a larger number of drug resistant fluke in Australia [42].

### Clonal isolates will advance our understanding of drug resistance in *Fasciola hepatica*

A lack of understanding of the specific mechanisms involved in drug resistance in *F. hepatica* raises several important questions about the origins, heritability and spread of drug resistance alleles in liver fluke populations. The clonal isolates reported here allow, for the first time, genetic crossing experiments between TCBZ-S and TCBZ-R isolates and supports subsequent genome mapping approaches that are crucial to addressing these complex questions in future [21, 43, 44]. A lack of population structure and high gene flow in *F. hepatica* populations has revealed that TCBZ resistance has the potential to spread rapidly [36]. The capacity for snails infected with a single miracidium to shed large numbers of clonal metacercariae raises the possibility for aggregation of resistant genotypes on pasture which in turn may lead to infection of the definitive host with large numbers of drug-resistant parasites [25]. Thus, the clonal amplification of *F. hepatica* genotypes within *G. truncatula* demonstrated here highlights the potential for drug resistance alleles to accumulate rapidly within liver fluke populations.

Importantly, at a practical level our work provides valuable insights into interpretation of drug efficacy studies given the genetic diversity inherent in liver fluke populations. In the case of *Fh*LivS1 and *Fh*LivS3, critical tests using the population of parasites from which these drug sensitive clonal isolates were derived, showed a TCBZ efficacy of 97% (Table 1). This means that even in highly TCBZ susceptible populations a proportion of live, egg producing adult parasites survive and are retained in the liver post-treatment. This is in contrast to our observations here with clonal susceptible parasites where only one genotype is present and complete removal of parasites was seen post-TCBZ treatment (Table 2). It is not known whether the parasites that survive treatment are TCBZ-susceptible parasites or if they represent drug-resistant parasites within a population where TCBZ resistance alleles are at low frequency and where TCBZ-resistance is emerging. It is interesting to note that the TCBZ-resistant isolate *Fh*LivR2, was derived from a population of parasites that, based on rigorous faecal egg count reduction test (FECRT) evaluation, was designated as TCBZ-susceptible [43]. This highlights the complexities of interpreting the FECRT and identifies the need for sensitive, molecular-based tests to determine the emergence of drug resistance in the field. Clonal isolates, such as those reported here will significantly enhance our ability to map drug resistance markers by integrating genetic crossing experiments with genome-wide bioinformatics approaches [20].

## Conclusions

A genetic clonal expansion occurs within *G. truncatula*, highlighting the potential for amplification of drug resistant genotypes of *F. hepatica*. Variation in the weight of parasites within and between clonal isolates and when comparing isolates that are either susceptible or resistant to TCBZ represents inherent variation in liver fluke and cannot be attributed to their resistance or susceptibility traits. Clonal isolates will facilitate genetic mapping of drug resistance markers.

## Abbreviations

AHVLA: Animal Health Veterinary Laboratory Agency
APHA: Animal and Plant Health Agency
ELISA: Enzyme-linked immunosorbent assay
epg: Eggs per gram
FEC: Faecal egg count
FECRT: Faecal egg count reduction test
Fh: *Fasciola hepatica*
*Fh*Liv: Clonal isolate of *Fasciola hepatica*
MLG: Multilocus genotype
NEJ: Newly excysted juvenile
PGP: P-glycoprotein
PM: *Post-mortem*
PP: Percentage positivity
T: Treated with 10 mg/kg triclabendazole 10 days prior to PM
TCBZ-: No treatment with 10 mg/kg triclabendazole
TCBZ: Triclabendazole
TCBZ+: Treatment with 10 mg/kg triclabendazole
TCBZ-R: Triclabendazole-resistant
TCBZ-S: Triclabendazole-susceptible
wpi: Weeks post-infection
